# Interictal EEG and ECG for SUDEP Risk Assessment: A Retrospective Multicenter Cohort Study

**DOI:** 10.3389/fneur.2022.858333

**Published:** 2022-03-18

**Authors:** Zhe Sage Chen, Aaron Hsieh, Guanghao Sun, Gregory K. Bergey, Samuel F. Berkovic, Piero Perucca, Wendyl D'Souza, Christopher J. Elder, Pue Farooque, Emily L. Johnson, Sarah Barnard, Russell Nightscales, Patrick Kwan, Brian Moseley, Terence J. O'Brien, Shobi Sivathamboo, Juliana Laze, Daniel Friedman, Orrin Devinsky, The MS-BioS Study Group

**Affiliations:** ^1^Department of Psychiatry, New York University Grossman School of Medicine, New York, NY, United States; ^2^Neuroscience Institute, New York University Grossman School of Medicine, New York, NY, United States; ^3^Tandon School of Engineering, New York University, New York, NY, United States; ^4^Johns Hopkins University School of Medicine, Baltimore, MD, United States; ^5^Department of Medicine (Austin Health), The University of Melbourne, Heidelberg, VIC, Australia; ^6^Comprehensive Epilepsy Program, Department of Neurology, Austin Health, Heidelberg, VIC, Australia; ^7^Department of Neuroscience, Central Clinical School, Monash University, Melbourne, VIC, Australia; ^8^Department of Neurology, Alfred Health, Melbourne, VIC, Australia; ^9^Department of Neurology, The Royal Melbourne Hospital, Melbourne, VIC, Australia; ^10^Department of Medicine, St. Vincent's Hospital, The University of Melbourne, Fitzroy, VIC, Australia; ^11^Division of Epilepsy and Sleep, Columbia University, New York, NY, United States; ^12^Yale University School of Medicine, New Haven, CT, United States; ^13^Department of Neurology, New York University Grossman School of Medicine, New York, NY, United States; ^14^Department of Medicine, The Royal Melbourne Hospital, The University of Melbourne, St Vincent's Hospital Melbourne, Melbourne, VIC, Australia; ^15^Clinical Development Neurocrine Biosciences Inc., San Diego, CA, United States; ^16^Comprehensive Epilepsy Center, New York University Langone Health, New York, NY, United States

**Keywords:** SUDEP, biomarker, machine learning, EEG, ECG

## Abstract

**Objective:**

Sudden unexpected death in epilepsy (SUDEP) is the leading cause of epilepsy-related mortality. Although lots of effort has been made in identifying clinical risk factors for SUDEP in the literature, there are few validated methods to predict individual SUDEP risk. Prolonged postictal EEG suppression (PGES) is a potential SUDEP biomarker, but its occurrence is infrequent and requires epilepsy monitoring unit admission. We use machine learning methods to examine SUDEP risk using interictal EEG and ECG recordings from SUDEP cases and matched living epilepsy controls.

**Methods:**

This multicenter, retrospective, cohort study examined interictal EEG and ECG recordings from 30 SUDEP cases and 58 age-matched living epilepsy patient controls. We trained machine learning models with interictal EEG and ECG features to predict the retrospective SUDEP risk for each patient. We assessed cross-validated classification accuracy and the area under the receiver operating characteristic (AUC) curve.

**Results:**

The logistic regression (LR) classifier produced the overall best performance, outperforming the support vector machine (SVM), random forest (RF), and convolutional neural network (CNN). Among the 30 patients with SUDEP [14 females; mean age (SD), 31 (8.47) years] and 58 living epilepsy controls [26 females (43%); mean age (SD) 31 (8.5) years], the LR model achieved the median AUC of 0.77 [interquartile range (IQR), 0.73–0.80] in five-fold cross-validation using interictal alpha and low gamma power ratio of the EEG and heart rate variability (HRV) features extracted from the ECG. The LR model achieved the mean AUC of 0.79 in leave-one-center-out prediction.

**Conclusions:**

Our results support that machine learning-driven models may quantify SUDEP risk for epilepsy patients, future refinements in our model may help predict individualized SUDEP risk and help clinicians correlate predictive scores with the clinical data. Low-cost and noninvasive interictal biomarkers of SUDEP risk may help clinicians to identify high-risk patients and initiate preventive strategies.

## Introduction

Sudden unexpected death in epilepsy (SUDEP) is the leading cause of epilepsy-related mortality (>3, 000 deaths/year in the US), and the second leading neurological cause of lost patient life-years (1–4). Usually, SUDEP occurs during sleep and death is unwitnessed (5, 6). Treatment-resistant patients have the highest SUDEP risk. There are currently no validated biomarkers to predict individual SUDEP risk. Risk reduction strategies include convulsive seizure control and nocturnal monitoring (3, 7, 8). Generalized tonic–clonic seizure (GTCS) frequency and nocturnal convulsions are leading SUDEP risk factors (9–12). Supervision during sleep may reduce SUDEP risk. Prolonged postictal EEG suppression (PGES) is a potential SUDEP biomarker (13–16) but requires epilepsy-monitoring unit admission. The cost and potential risk limit PGES, which is available in <5% of epilepsy patients (4). Furthermore, nonseizure SUDEP cases can occur (17), supporting, the need for interictal biomarkers of SUDEP risk.

Resting-state functional MRI (fMRI) (18, 19) may detect activity in brainstem cardiopulmonary centers and their cortical connections. Altered resting-state functional connectivity between cortical-subcortical brain regions is implicated in SUDEP (20). Large-scale functional brain networks may alter neuronal dynamics, detectable on interictal EEG. Furthermore, heart rate variability (HRV) is a biomarker of autonomic dysfunction and potentially SUDEP risk (21–25). We recently demonstrated altered HRV in SUDEP cases compared with the matched controls (23). Combining both the EEG and ECG measures might improve the efficacy of prediction models. Critically, interictal EEG and ECG are low cost and widely available.

Machine learning has strong predictive power and promising potentials for applications of medical and neurological disorders (26–28), and has been increasingly applied to clinical diagnosis and prognosis. Machine learning methods are used for EEG-based seizure detection (29), but infrequently to predict SUDEP risk (30–33). We applied machine learning methods to analyze interictal EEG and ECG recordings to assess individualized SUDEP risk. We aimed to identify biomarkers of SUDEP risk and correlate the classification score with clinical variables. We conducted data-driven SUDEP classification and survival analyses and verified the machine-learning models using a retrospective multicenter data cohort.

## Materials and Methods

### Study Population and Cohort

This multicenter, retrospective, case–control study identified SUDEP cases among patients admitted to eight tertiary epilepsy monitoring units (EMUs) of the MS-BioS Study Group, including the Royal Melbourne Hospital, Austin Hospital, St. Vincent's Hospital, Melbourne, Australia; NYU Langone Health, NY Presbyterian Hospital/Columbia University, New York; University of Cincinnati, Cincinnati; Yale New Haven Hospital, New Haven; and Johns Hopkins Medical Center, Baltimore). Patients underwent video EEG monitoring (VEM) with ≥ 21 scalp electrodes using the 10–20 system and lead II of a standard 12-lead ECG.

Each center identified patients aged 6 months to 65 years with ≥1 electroclinical seizure recorded over a 2–11-year consecutive period (25). All the patients were followed for ≥5 years. Epilepsy-related deaths were reviewed with available records, medical examiner/coronial, and autopsy findings to determine the cause of death. We included definite and probable SUDEP cases based on the current criteria (34).

For each SUDEP case, two living epilepsy controls were matched according to admission age (±4 years), sex, and EMU admission year (±1 year) from the EMU cohort at each center. Epilepsy controls had documented contact in the medical record within 6 months of screening or were identified as not deceased from national death records.

### Demographical and Clinical Data

For all the cases, demographical and clinical data including epilepsy and seizure classification; seizure frequency; the age of onset; antiseizure medications (ASMs), epilepsy surgery or neuromodulation; other medications; and medical history (e.g., cardiovascular and psychiatric disorders) were obtained from medical record review at EMU admission.

### Recording Selection

Electroencephalogram sampling rates ranged between 256 and 512 Hz. We identified 10-min interictal segments from nonrapid eye movement (NREM) sleep and 10-min segment from wakefulness during VEM for each case, typically from the first 24 h of VEM. For most of the studies, we were limited to review of archived EEG which did not include the complete recording and just snippets that were selected for archiving by the clinician. Sleep segments were chosen at random that preceded at least 1 h before or after a nonconvulsive seizure or 6 h before or after a convulsive seizure. During wakefulness, EEG was collected when subjects were free of muscle and movement artifact. These artifact-free segments were chosen by a well-trained epileptologist or a research scientist, but were done without the knowledge of any hypothesis. We excluded the immediate postictal period, which was defined as ≥6 h following tonic–clonic seizures (TCSs) and ≥1 h for all other seizure types. EEG and ECG recordings were converted from the proprietary formats to the ASCII format using Persyst 13 (Prescott, Arizona, USA). Similarly, 10-min interictal segments of stable ECG were selected from both the NREM sleep and wakefulness (exactly the same time of EEG recordings) for each subject (25).

### Offline EEG and ECG Feature Computation

For each EEG group signal, we performed bandpass filtering (1–100 Hz) and then calculated the relative power ratio at six frequency bands: delta (1–4 Hz), theta (4–8 Hz), alpha (8–15 Hz), beta (15–30 Hz), low gamma (30–50 Hz), and high gamma (50–100 Hz). The spectral power was calculated using fast Fourier transform (FFT) with a multitaper estimator on the entire 10-min recordings. To account for the measurement variability between subjects or centers, we used the relative power percentage and power ratio to calibrate. For instance, the feature derived from the low gamma band was defined as:


$$\begin{array}{l} \text{Relative\_low\_gamma\_power}=\frac{\text{low gamma power }\left(30-50\text{ Hz}\right)}{\text{broad band power }\left(1-100\text{ Hz}\right)},\\ \text{Low\_gamma\_power\_ratio}=\frac{\text{relative\_low\_gamma\_power }\left(\text{SLEEP}\right)}{\text{relative\_low\_gamma\_power }\left(\text{WAKE}\right)} \end{array}$$

**Figure 1:**
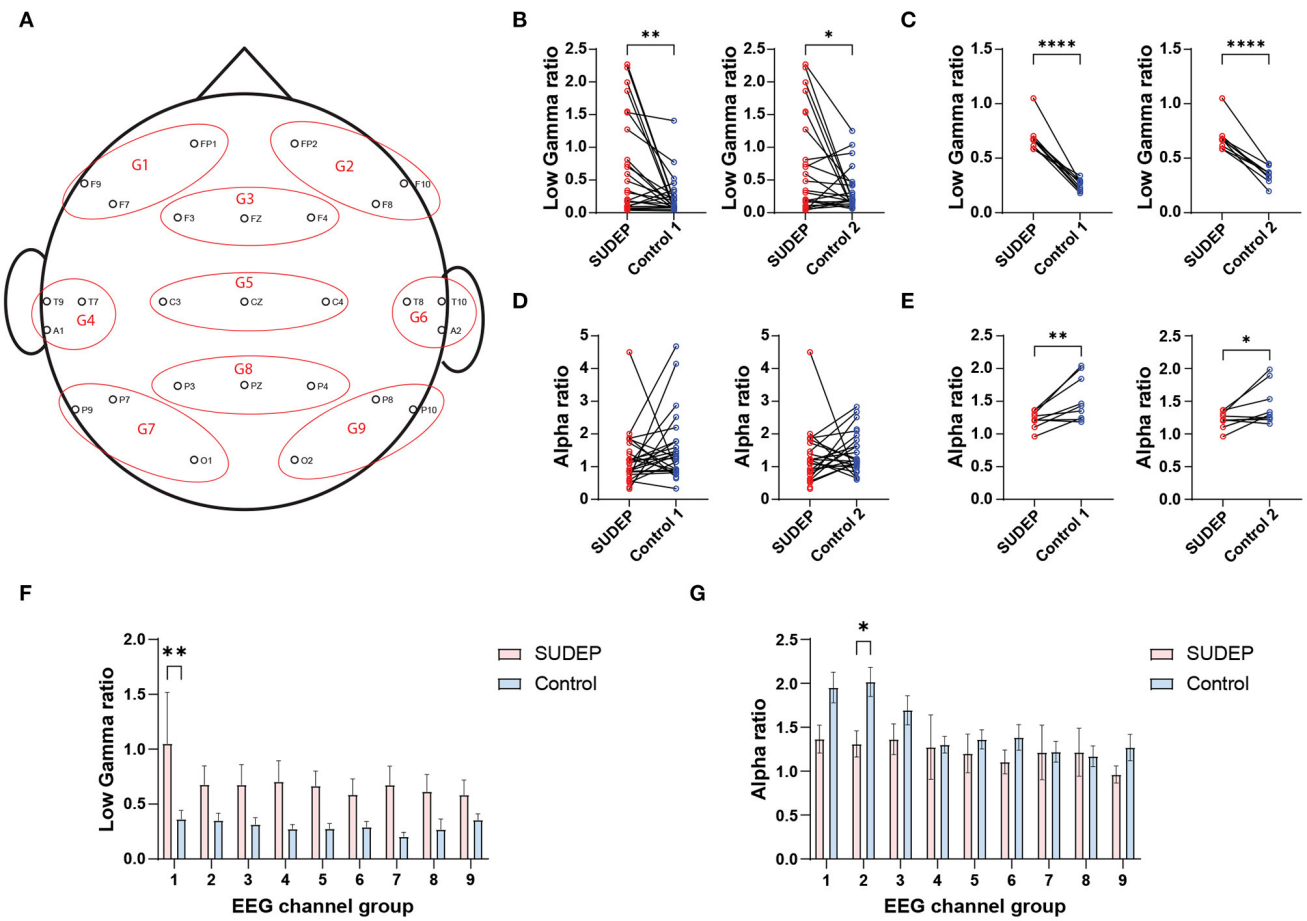
**(A)** Clustering scalp electroencephalography (EEG) electrodes (10–20 International System) into nine channel groups (G1–G9). **(B)** Comparison of channel-averaged EEG low gamma sleep/wake power ratios between SUDEP Patients and age-matched living epilepsy controls (SUDEP vs. control 1, **p = 0.0033, paired *t*-test; SUDEP vs. control 2, *p = 0.0251). **(C)** Comparison of subject-averaged EEG low gamma sleep/wake power ratios between SUDEP patients and age-matched living epilepsy controls (****, *p* < 0.0001, paired *t*-test). **(D,E)** Similar to panels **(B,C)** except for the alpha band [panel **(D)**: n.s., *p* = 0.258 and *p* = 0.719; panel **(E)**: **p = 0.009 and *p = 0.039, paired *t*-test]. **(F)** Comparison of EEG low gamma sleep/wake power ratios between SUDEP patients and age-matched controls in nine EEG channel groups (**, *p* = 0.0012, two-way ANOVA test; error bar denotes SEM). **(G)** Similar to panel F, except for the alpha band (**, *p* = 0.048, two-way ANOVA test).

We used the EEG power ratio for the between-subject EEG power calibration purpose. To reduce the feature dimensionality and avoid overfitting, we clustered scalp EEG electrode channels into nine groups (Figure 1A and Supplementary Methods). In total, we had 6 × 9 = 54 (frequency × group) power ratio features for an individual subject. To further reduce the number of power ratio features, we ranked these EEG features using a linear support vector machine (SVM) classifier (i.e., polynomial kernel with degree 1). The SVM weights associated with individual features determined their relative importance (35). We further selected the most discriminative features (“*alpha*_*power*_*ratio*” and “*low*_*gamma*_*power*_*ratio*”) among specific channel groups (Figures 1B–E).

To analyze ECG recordings, we calculated a set of standardized linear (time and frequency-domain) and nonlinear ECG features (a total of 24 ECG-HRV features per subject, see Supplementary Methods and Supplementary Figure S1) and then optimized features. For the sake of feature calibration, we again calculated each HRV feature's sleep/wake ratio, so that the actual feature was dimensionless. While using the EEG and ECG feature ratios, we did not impose any statistical independence criterion and resorted on an unbiased feature selection procedure. The flowchart of EEG/ECG data analytics is shown in Supplementary Figure S2.

### Machine Learning Methods

We tested multiple standard machine learning methods, including the logistic regression (LR) classifier, linear SVM, and random forest (RF). In all the classification methods, the binary label 0/1 represents the non-SUDEP/SUDEP identity in this retrospective study. To alleviate overfitting, simpler models were preferred because of small sample size.

### Feature Selection

In offline classification task, we considered frequency-specific power ratio features in sleep and wake EEG; and sleep ECG-HRV features. For combined EEG and ECG features, we conducted two systematic approaches to select features. We trained all features with an LR, SVM, and RF classifiers with an *L*_1_ norm sparsity constraint, and identified relevant features associated with nonzero regression coefficients after 1,000 runs. Next, we retrained the classifier with fewer features without the *L*_1_ norm.

To assess online SUDEP risk, we adopted a sliding window to extract features of the EEG during sleep and then applied the standard machine learning classifiers to the data from each moving window. We employed a parametric CNN architecture (Supplementary Figure S3) with one-dimensional convolutional filters to model the power, frequency, and phase relationships between EEG channels (36).

It is noted that we did not include any clinical measurement as the predictive features for two reasons. First, we would provide an unbiased analysis without using the clinical diagnostics; instead, we only correlated the predicted risk probability with the clinical variables in the *post-hoc* analysis. Second, missing data of clinical variables were present in many subjects in this study.

### Survival Analysis

The clinical variable EMU-to-SUDEP interval (ranged between 0.5 and 10 years) defined the time from EMU recording to the SUDEP incident. To characterize the EMU-to-SUDEP risk in the SUDEP group, we used Cox proportional hazards model with an imposed an *L*_1_ norm sparsity constraint: λ(*t*|*X*_*i*_) = λ_0_exp(*X*_*i*_β), where λ_0_ is baseline hazard, *X*_*i*_ are covariates for *i*-th subject, and β is the regression parameter. The survival analysis was aimed to predict the EMU-to-SUDEP interval with both EEG and ECG features. We used the LASSO method to select the candidate EEG and ECG features (37). Because of missing data in some SUDEP patients, no other clinical variable was used in the survival analysis. The sparsity constraint on β improved the survival model generalization. The regularization parameter α was estimated by a grid search followed by five-fold cross-validation. We used the concordance index (range 0–1) as the goodness-of-fit assessment, where 1 implies the perfect prediction.

### Performance Evaluation

The sensitivity, specificity, accuracy, and the area under the receiver operating characteristic (AUC) curve were calculated for all the machine learning classifiers. Median AUC and IQR (25 and 75% percentiles) were calculated using five-fold cross-validation with 1, 000 random repeats. In leave-one-center-out prediction, we used the data from 7 centers to train the model and one center to test the model.

### Statistical Analysis

Data were analyzed with custom software written in MATLAB and Python. Statistical significance of parametric or nonparametric tests used in all analyses was set at *P* < 0.05. Multiple comparisons were corrected using Bonferroni correction. To promote rigor and reproducibility, the data analytic software is shared online (https://github.com/aaronh314/SUDEP).

## Results

### Study Population

Table 1 presents demographical and clinical data on the study population (for individual center data, see Supplementary Table S1) (25). Data for cases and controls were collected at EMU admission, except for surgical intervention(s) and Engel outcome, collected at last follow-up. The interval between VEEG and SUDEP was 0.5–10 years.

**Table 1:** Demographical and clinical characteristics of the study population [modified from Ref. (25)].

**Characteristic**	**SUDEP cases** **(*n*=30)**	**Living epilepsy controls** **(*n*=58)**	***P-*value**
Age—yr, median [IQR]	34 [24, 40]	34 [25, 40]	1.0
Male gender, *n* (%)	16 (53.3%)	29 (50%)	0.176
Race, *n* (%)			0.447
White	25 (83.3%)	43 (74.1%)	
Black/African American	3 (10%)	6 (10.3%)	
Asian	1 (3.3%)	3 (5.2%)	
Other	1 (3.3%)	4 (6.9%)	
Unknown	0 (0%)	2 (3.4%)	
Epilepsy classification, *n* (%)			0.527
Focal	25 (83.3%)	48 (82.8%)	
Generalized	4 (13.3%)	9 (15.5%)	
Combined focal and generalized	1 (3.3%)	1 (1.7%)	
Unknown	0 (0%)	0 (0%)	
Etiology, *n* (%)			0.583
Structural/Metabolic	15 (50%)	24 (41.4%)	
Genetic/Presumed Genetic	3 (10%)	8 (13.8%)	
Unknown	12 (40%)	26 (44.8%)	
Antiseizure medications on admission, *n* (%)			0.847
None	0 (0%)	2 (3.4%)	
Monotherapy	4 (13.3%)	11 (19%)	
Polytherapy (≥2)	26 (86.7%)	45 (77.6%)	
Age of onset ^‡^–yr, median [IQR]	10 [2, 16]	12 [3, 21]	0.571^¶^
Disease duration—yr, median [IQR]	17 [12, 33]	14 [5, 29]	0.083^¶^
EMU to SUDEP time—yr, median [IQR]	2 [4, 6]	n/a	n/a
Lifetime tonic-clonic seizure (TCS) frequency^§^, *n* (%)			
None	3 (10%)	15 (25.9%)	0.231^*^
≥1, but <6	3 (10%)	15 (25.9%)	0.231^*^
≥6, but <50	5 (16.7%)	5 (8.6%)	0.273^*^
≥50	7 (23.3%)	2 (3.4%)	0.016^*^
Unknown	12 (40%)	21 (36.2%)	n/a
Outcome of surgical intervention, *n* (%)			
Engel I	1 (3.3%)	8 (13.8%)	0.264^*^
Engel II	1 (3.3%)	5 (8.6%)	0.624^*^
Engel III	3 (10%)	2 (3.4%)	0.566^*^
Engel IV	3 (10%)	1 (1.7%)	0.324^*^
Unknown	4 (13.3%)	1 (1.7%)	n/a
Cardiovascular disease, *n* (%)			
Hypertension	3 (10%)	4 (6.9%)	0.696
Cardiac arrhythmia	1 (3.3%)	0 (0%)	0.356
Structural heart disease	3 (10%)	0 (0%)	**0.042**
Sleep apnea	0 (0%)	1 (1.7%)	1.0
Psychiatric comorbidity, *n* (%)			
Anxiety disorder	0 (0%)	7 (12.1%)	**0.047**
Depression	2 (6.7%)	16 (27.6%)	**0.015**
Medication for psychiatric disorder, *n* (%)			
Antipsychotic	3 (10%)	2 (3.4%)	0.343

*IQR, interquartile range; EMU, epilepsy monitoring unit*.

‡ *Age of onset unknown in two (3.4%) epilepsy controls*.

¶ *P-value calculated with a two-sample Wilcoxon rank-sum test*.

§ *Includes both focal-to-bilateral tonic-clonic seizures (TCSs) and generalized tonic-clonic seizures (GTCSs)*.

* *Statistical significance corrected p-value following Holm-Bonferroni adjustment for multiple comparisons. Bold font indicates statistical significance (p < 0.05)*.

We analyzed EEG recordings from 30 patients with SUDEP and 58 living epilepsy controls. A subset of 83 subjects had 10-min interictal sleep EEG recordings (in one SUDEP case and four controls, interictal EEG recordings during sleep were unavailable). Furthermore, 76 subjects (26 SUDEP and 50 controls) from this subset had both the interictal sleep and wake EEG recordings and 70 of these 76 subjects had clean ECG recordings.

### Feature Selection, Classification, and Survival Analysis

We calculated three sets of features: (i) EEG frequency-power ratios during sleep and wake states; (ii) ECG-HRV features, and (iii) sliding window-based sleep EEG features only. For each set, we ranked individual features to compare classification utility. From the combined features (i) and (ii), we identified an optimal subset and compared cross-validated accuracy (Supplementary Figure S4). The optimal set of EEG + ECG features varied between 2 and 5 and we reported the statistics using 3. We trained classifiers using single or combined features separately. For the *L*_1_ regularized LR classifier using features (i) + (ii), the significant coefficients include alpha power ratio, high gamma power ratio, and HRV lf/hf power ratio (Supplementary Figure S5).

**Figure 2:**
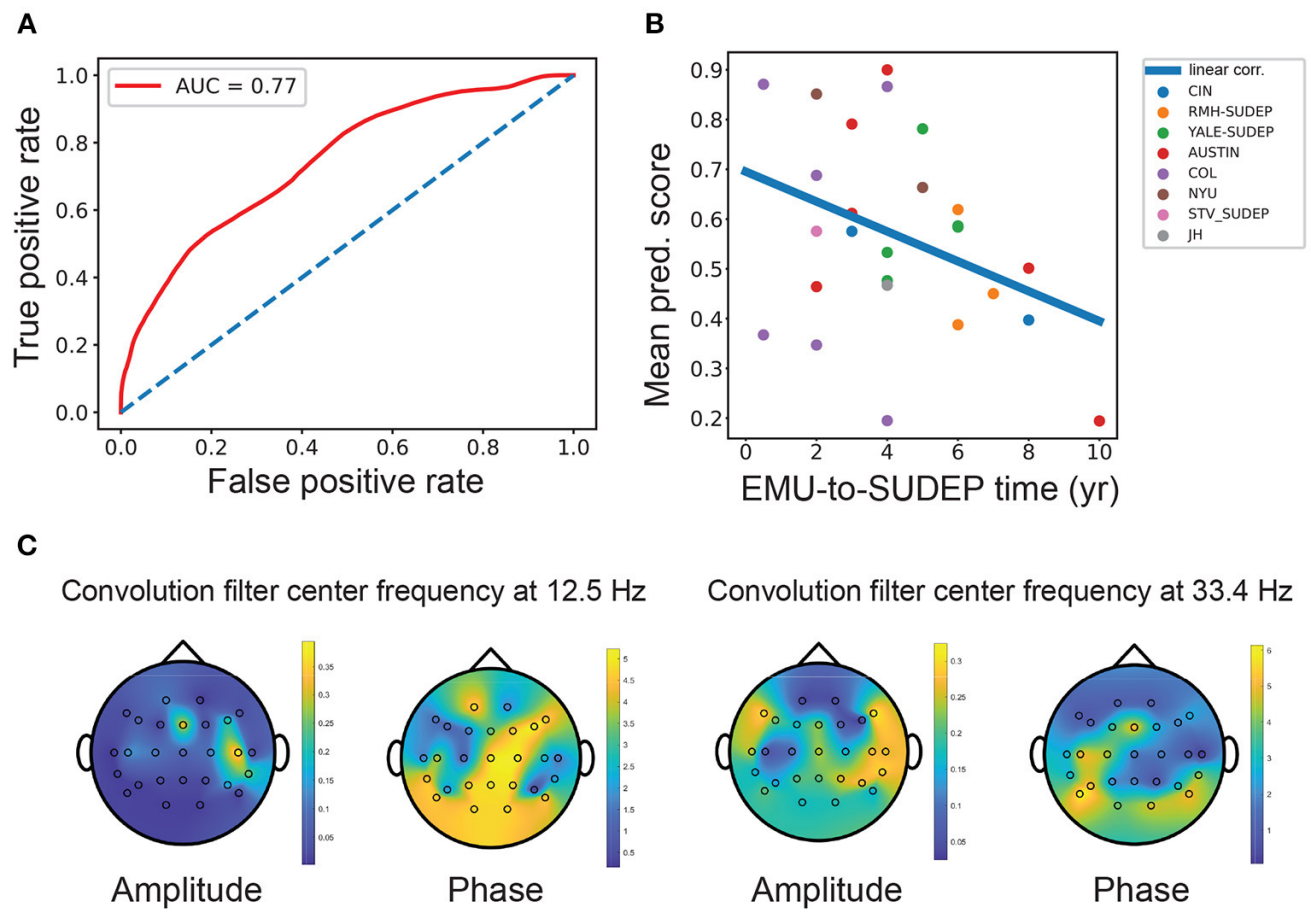
**(A)** Mean receiver operating characteristic (ROC) curve [mean area under the curve (AUC) = 0.77, interquartile range (IQR): 0.73–0.80; LR classifier] obtained from SUDEP vs. non-SUDEP classification based on combined EEG and ECG features. Diagonal line shows the chance level (AUC = 0.5). **(B)** The mean SUDEP prediction score correlated negatively with the EMU-to-SUDEP time among the SUDEP group (Pearson's correlation ρ =-0.38, *n* = 26). Color-coded points represent patients from 8 different centers. **(C)** Visualization and projection of two pairs of convolutional filters in the CNN onto the brain topographies of spatial patterns. The spatial patterns of “amplitude map” indicate the importance at specific channels, whereas the spatial patterns of “phase shift map” indicate the relative phase lagging.

In offline classification, the LR and SVM classifiers achieved comparable or nonsignificantly different results. The best five-fold randomized cross-validated AUC [median 0.77, interquartile range (IQR) 0.73–0.80; 1, 000 Monte Carlo runs] was based on the LR classifier (Figure 2A and Table 2). Combining EEG and ECG features slightly improved performance for most classifiers, suggesting features are complementary. The low gamma power ratio was significantly higher in the SUDEP patients (especially for EMU-to-SUDEP <5 years) than controls; most significant in temporal lobes (i.e., EEG groups 4–6; Figure 1F). The alpha power ratio was significantly lower in SUDEP cases vs. controls (Figure 1G). Therefore, low gamma and alpha power ratio from specific regions were the most discriminative SUDEP risk features. As a comparison, we also trained ML classifiers using ECG features alone (*n* = 70, based on the same feature selection procedure). After feature ranking, we selected the most discriminative four ECG-HRV features (“lfnu,” “hfnu,” “sd1,” and “ratio_sd2_sd1”) for classification analysis, and the cross-validated AUC results were as follows: LR median 0.65 (IQR 0.61–0.69), SVM median 0.55 (IQR 0.47–0.62), and RF median 0.58 (IQR 0.52–0.65).

**Table 2:** Comparison of model performance [median interquartile range (IQR)] in five-fold cross-validation.

**Feature set**	**# SUDEP + control**	**Model**	**AUC**	**Sensitivity**	**Specificity**	**Accuracy**
(i) + (ii) (i) (iii)	70 = 24 + 46 76 = 26 + 50 83 = 29 + 54	LR	0.77 [0.73, 0.80] 0.75 [0.73, 0.78] 0.64 [0.60, 0.67]	0.63 [0.59, 0.67] 0.64 [0.60, 0.70] 0.73 [0.65, 0.79]	0.69 [0.66, 0.72] 0.68 [0.64, 0.72] 0.39 [0.29, 0.49]	0.65 [0.62, 0.69] 0.66 [0.64, 0.69] 0.55 [0.50, 0.59]
(i) + (ii) (i) (iii)	70 = 24 + 46 76 = 26 + 50 83 = 29 + 54	SVM	0.74 [0.70, 0.78] 0.74 [0.68, 0.78] 0.61 [0.53, 0.66]	0.65 [0.57, 0.71] 0.70 [0.59, 0.79] 0.73 [0.59, 0.79]	0.64 [0.58, 0.71] 0.59 [0.53, 0.64] 0.40 [0.29, 0.49]	0.64 [0.60, 0.68] 0.63 [0.58, 0.68] 0.53 [0.50, 0.57]
(i) + (ii) (i) (iii)	70 = 24 + 46 76 = 26 + 50 83 = 29 + 54	RF	0.71 [0.66, 0.76] 0.61 [0.57, 0.66] 0.59 [0.54, 0.64]	0.67 [0.62, 0.72] 0.58 [0.54, 0.62] 0.52 [0.45, 0.59]	0.66 [0.59, 0.71] 0.61 [0.54, 0.66] 0.63 [0.58, 0.69]	0.66 [0.62, 0.70] 0.59 [0.55, 0.63] 0.57 [0.53, 0.62]
(iii)	83 = 29 + 54	CNN	0.60 [0.57, 0.64]	0.45 [0.38, 0.52]	0.66 [0.59, 0.73]	0.55 [0.52, 0.57]

We performed simulated “online” classification based on sleep EEG recordings and found classification accuracy degraded compared with the offline classification. We optimized the window size and features (Supplementary Figure S6), and achieved the best five-fold cross-validated AUC 0.64 from the LR classifier. The CNN achieved a median AUC 0.60 (IQR 0.57–0.64), sensitivity 0.45 (IQR 0.38–0.52), specificity 0.66 (IQR 0.59–0.73), and accuracy 0.55 (IQR 0.52–0.57). The simple LR classifier achieved the overall best AUC performance. To test the model stability in sleep EEG signal nonstationarity, we trained the LR classifier with the first half of sleep EEG and tested the second half; results were comparable, mean cross-validated AUC 0.74 (Supplementary Figure S7).

In the leave-one-center-out prediction setting, only data from seven centers were used to train the model, followed by the validation on the held-out data from the remaining one center. In this case, the LR classifier had mean AUC of 0.734 (minimum: 0.5, maximum: 1.0) and mean accuracy of 0.55 (minimum: 0.25, maximum: 0.9) (Supplementary Table S2). Thus, the mean AUC result was comparable with the standard five-fold cross-validation analysis, suggesting moderate generalization at different settings.

In survival analysis within the SUDEP at-risk patients, we obtained the averaged concordance index of 0.687 from five-fold cross-validation (minimum: 0.44, maximum: 0.90) from the regularized Cox proportional hazard model with a sparsity constraint. This result was comparable to the SUDEP group classification accuracy. The significant coefficients include relative alpha power, the mean heart rate (HR) and the low frequency (LF) power of HRV.

### Interpretation of Classification Results

The AUC statistic can assess diagnostic ability with dichotomous outcomes. Our best offline AUC performance was 0.74–0.77, acceptable considering the small sample size (38). Clinically, the sample size is crucial to interpret statistical significance (39, 40). Among the patients with SUDEP, the median prediction score showed a negative trend by correlating with the EMU-to-SUDEP time (Figure 2B, Pearson's correlation ρ =-0.38, *n* = 26, *p* = 0.054, SVM; ρ = −0.36, *p* = 0.07, LR). Patients with SUDEP with short latency (EMU-to-SUDEP time <5 years) were more accurately classified than those with long latency (≥5 years). Furthermore, epilepsy patients with SUDEP with EMU-to-SUDEP time >7 years were misclassified (i.e., treated as false negatives), suggesting that they were closer to living epilepsy controls than other patients with relatively lower SUDEP risk. Epilepsy controls misclassified by LR had low/high gamma power higher in all the channels. In addition, 16.7% of the false positive group, had depression and 6.7% of the true positive group had depression; 33% of the true negative group had depression. A third of the false positive group had Developmental Delay/Static Encephalopathy, and 40% of the true positive group had Developmental Delay/Static Encephalopathy; 18% of true negatives had Developmental Delay/Static Encephalopathy (Supplementary Table S3).

The CNN can extract informative spatio-spectral features from multichannel EEG data. As the epileptic brain often shows synchronized sleep EEG patterns across brain regions, convolutional filters (2–4 pairs) in the CNN aimed to capture the cross-spectral (amplitude and phase) features between EEG electrodes. To help visualize these filters, we projected the respective amplitude and phase shift at the same central frequency onto the brain topographies of spatial patterns. The spatial patterns of “amplitude map” indicates the importance at specific channel, whereas the spatial patterns of “phase shift map” indicates the relative phase lagging (Figure 2C and Supplementary Methods). At the low frequency (~12.5 Hz), the peak amplitude was grouped around the frontal-temporal lobe electrodes, where the frontal electrodes had a phase lead compared with the central/parietal/occipital electrodes. At the gamma frequency (~33.4 Hz), the peak amplitude was around the temporal lobe, where the occipital electrodes had a phase lag with respect to other electrodes.

## Discussion

This study demonstrates that machine learning tools using interictal EEG and ECG can help distinguish high-risk from low-risk patients with SUDEP. Our feature selection procedure identified key interictal EEG or ECG-HRV features in assessing individual SUDEP risk. The CNN extracted complex nonlinear spatiospectral features in sleep EEGs. We plan to refine our model on prospectively ascertained SUDEP and control cohorts. Development of SUDEP biomarker-informed preventive strategies will be the subject of future investigation.

Recently, it has been suggested that ictal biomarkers for PGES/SUDEP based on the seizure generation and termination (41, 42). It is possible that the ictal episodes may carry the most predictive power for SUDEP risk assessment. However, it remains uncertain if SUDEP risk can be predicted from interictal epileptiform discharges (IEDs) in sleep (43, 44). We identified robust differences in EEG sleep/wave power ratio features in low gamma, high gamma, and alpha bands between SUDEP and control patients. The effect was pronounced over frontotemporal regions in the scalp EEG recordings, which may correlate with seizure-onset regions; however, detailed investigations are still required to unravel their relationship. An intracranial EEG study has shown that gamma oscillations precede seizure onset zone IEDs (45); relative high sleep/wake gamma power ratio may reflect the more frequent IED activity in the SUDEP group. Cross-frequency coupling (e.g., delta-gamma phase-amplitude coupling) may improve prediction (46). Future studies by integration of multistage and multimodal neuroimaging might reveal mechanisms of SUDEP. Furthermore, systematical investigations of the EEG relationship between ictal seizure episodes and interictal episodes will be valuable to understand their contributions to SUDEP. Challenges remain for collection such dataset and development of proper data analytics.

Abnormalities in HRV are linked to sudden cardiac death and SUDEP risk. Patients with drug-resistant epilepsy have more autonomic dysfunction, lower awake HRV and greater variances between wake and sleep states than drug-responsive patients (23). We found reduced LF HRV power was reduced in SUDEP cases and predicted SUDEP latency (25). LF reflects sympathetic and parasympathetic activity. Combining EEG and ECG, it improved predictive power over EEG. Analyses combining interictal EEG and new ECG features may improve individual SUDEP prediction (47). Large sample size can greatly improve machine learning.

Advancing machine learning models of SUDEP risk will benefit from integration of clinical, imaging, and interictal physiological data. Seizure pathways change on circadian and slower timescales (48), suggesting that analyzing multiple timescales may provide improve individualized SUDEP prediction, and potentially peak periods of SUDEP risk within circadian or ultradian cycles. Multimodal data fusion techniques can reveal how data modalities interact (49) and improve SUDEP prediction (50). Greater sample size would greatly improve clinical prognosis and decision (51–53).

Finally, it is also worth pointing out the limitations of this study. Our sample size was relatively small, which may lead to overfitting and limits interpretation. In addition, the selected EEG and ECG segments were relatively short, and did not cover multiple-day or multiple-session recording samples. We did not assess postictal EEG suppression nor correlate their features with interictal EEG-derived features. Although we have conducted leave-one-center-out validation, this study did not validate methods on an external patient population. Finally, our retrospectively acquired cohort prevents validating the classifiers using continuous video-EEG recordings.

## Conclusion

The results of this analysis suggest that machine learning methods can identify the risk of SUDEP in individual patients in a retrospective multicenter cohort study based on interictal EEG recordings. Combining interictal EEG and ECG-HRV features improves the classification performance. A simple LR classifier produces the overall best classification performance in randomized five-fold cross-validation and leave-one-center-out prediction settings. The CNN can potentially extract multichannel sleep EEG features used for online SUDEP risk assessment. Further studies are warranted to validate the results in larger and more diverse cohorts. The incorporation of other parameters associated with SUDEP (e.g., respiratory measurements and electrodermal activity) may improve the accuracy of models for individual prediction of SUDEP risk.

## Data Availability Statement

The raw data supporting the conclusions of this article will be made available by the authors, without undue reservation.

## Ethics Statement

The studies involving human participants were reviewed and approved by NYU Langone Health. The patients/participants provided their written informed consent to participate in this study.

## Author Contributions

ZC and OD designed the research. AH and GS analyzed the data. ZC drafted the manuscript. All authors edited the manuscript. All the authors contributed to the article and approved the submitted version of the manuscript.

## Funding

This study was funded by grants from the US National Institute of Neurological Disorders and Stroke (NINDS, R01-NS123928, R01-NS121776), the National Institute of Mental Health (NIMH, R01-MH118928) and National Science Foundation (NSF, CBET-1835000), the Multidisciplinary University Research Initiatives (MURI), the Centers for Disease Control and Prevention (CDC), the Finding a Cure for Epilepsy and Seizures (FACES), and the Oracle for Research Award. AH received a GLASS (Global Leaders and Scholars in STEM) funding from NYU Tandon School of Engineering. The funders had no role in the design and conduct of the study; collection, management, analysis, and interpretation of the data; preparation, review, or approval of the manuscript; and decision to submit the manuscript for publication.

## Conflict of Interest

ZC reports grants from the National Institutes of Health (NIH) and National Science Foundation (NSF) during the conduct of the study. ZC is also a founder and CEO of NeuroThX, LLC. ZC also received cloud computing resources supported by the Oracle for Research Award. DF receives salary support for consulting and clinical trial related activities performed on behalf of The Epilepsy Study Consortium, a non-profit organization. DF receives no personal income for these activities. NYU receives a fixed amount from the Epilepsy Study Consortium toward DF salary. Within the past two years, The Epilepsy Study Consortium received payments for research services performed by DF from: Alterity, Baergic, Biogen, BioXcell, Cerevel, Cerebral, Jannsen, Lundbeck, Neurocrine, SK Life Science, and Xenon. He has also served as a paid consultant for Neurelis Pharmaceuticals and Receptor Life Sciences. He has received travel support from the Epilepsy Foundation. He has received research support from NINDS, Epilepsy Foundation, Empatica, Epitel, UCB, Inc and Neuropace unrelated to this study. He serves on the scientific advisory board for Receptor Life Sciences. He holds equity interests in Neuroview Technology. He received royalty income from Oxford University Press. SB is supported by a Program Grant from the National Health and Medical Research Council of Australia (APP1091593). He reports grants from Eisai, UCB Pharma, and SciGen; has a patent for SCN1A licensed to various diagnostic companies with no financial return, a patent for PRRT2 gene licensed to Athena Diagnostics, and a patent for Diagnostic and Therapeutic Methods for Epilepsy and Mental Retardation Limited to Females (EFMR) licensed to Athena Diagnostics. PP is supported by an Early Career Fellowship from the National Health and Medical Research Council (APP1163708), the Epilepsy Foundation, The University of Melbourne, Monash University, Brain Australia, and the Weary Dunlop Medical Research Foundation. He has received speaker honoraria or consultancy fees to his institution from Chiesi, Eisai, the limbic, LivaNova, Novartis, Sun Pharma, Supernus, and UCB Pharma. He is an Associate Editor for Epilepsia Open. WD'S receives salary support from The University of Melbourne. He has received travel, investigator-initiated, scientific advisory board and speaker honoraria from UCB Pharma Australia & Global; investigator-initiated, scientific advisory board, travel and speaker honoraria from Eisai Australia & Global; advisory board honoraria from Liva Nova; educational grants from Novartis Pharmaceuticals, Pfizer Pharmaceuticals and Sanofi-Synthelabo; educational; travel and fellowship grants from GSK Neurology Australia, and honoraria from SciGen Pharmaceuticals. He has shareholdings in the device company EpiMinder. PK is supported by a Medical Research Future Fund from the National Health and Medical Research Council of Australia (MRF1136427) and the Victorian Medical Research Acceleration Fund. He reports grants and personal fees from Eisai, UCB Pharma, and LivaNova; reports grants from Zynerba, Biscayne, and GW Pharmaceuticals; and has received travel, speaker honoraria, or consultancy fees from Sun Pharmaceuticals, Supernus Pharmaceuticals, Novartis, and Eisai. BM is a paid employee at Clinical Development Neurocrine Biosciences Inc. BM has previously served as an advisory board member/consultant for Eisai and UCB Pharma and as a speaker for Eisai, LivaNova and UCB Pharma. He has previously received research support from GW Pharma, LivaNova, Nonin Medical, Inc, Sunovion and Xenon Pharmaceuticals. TO'B is supported by a Program Grant (APP1091593) and Investigator Grant (APP1176426) from the National Health and Medical Research Council of Australia and the Victorian Medical Research Acceleration Fund. He reports grants and consulting fees to his institution from Eisai, UCB Pharma, Praxis, Biogen, ES Therapeutics and Zynerba. SS is supported by a Bridging Postdoctoral Fellowship from Monash University (BPF20-3253672466) and the Victorian Medical Research Acceleration Fund. She reports salary support paid to her institution from Kaoskey and Optalert for clinical trial related activities; she receives no personal income for these activities. OD received grants from the NIH during the conduct of the study, and received funding from Finding A Cure for Epilepsy and Seizures (FACES) and has equity in Empatica.. The remaining authors declare that the research was conducted in the absence of any commercial or financial relationships that could be construed as a potential conflict of interest.

## Publisher's Note

All claims expressed in this article are solely those of the authors and do not necessarily represent those of their affiliated organizations, or those of the publisher, the editors and the reviewers. Any product that may be evaluated in this article, or claim that may be made by its manufacturer, is not guaranteed or endorsed by the publisher.

## Appendix

**Table A1:** MS-BioS study group.

**Name**	**Location**	**Role**	**Contribution**
Dale C. Hesdorffer, PhD	Columbia University, New York, United States	Principal investigator	Contributed to the acquisition of data.
Sylwia Misiewicz, Ed.M	Columbia University, New York, United States	Research coordinator	Contributed to the acquisition of data.
Lucy Mendoza, CCRP	University of Cincinnati, Cincinnati, United States	Research coordinator	Contributed to the acquisition of data.
