# Supplementary material for: Interictal EEG and ECG for SUDEP Risk Assessment: A Retrospective Multicenter Cohort Study
Source: Front Neurol. 2022 Mar 18;13:858333. doi: 10.3389/fneur.2022.858333 (PMC8973318; doi:10.3389/fneur.2022.858333)
Supplement: Supplementary file 1 [file Data_Sheet_1.docx]

**Supplementary Material**

**Supplementary Methods**

**EEG and ECG preprocessing and feature selection**

Since the EEG data were collected from multiple centers, the recording equipments were not uniform. To accommodate the variability and unify the format of EEG data, we divided EEG channels into 9 groups: G1 ('FP1', 'F7', 'F9'), G2 ('FP2', 'F8', 'F10'), G3 ( 'FZ', 'F3', 'F4'), G4 ('T7', 'T9', 'A1'), G5 ('T8', 'T10', 'A2'), G6 ('CZ', ' C3', 'C4'), G7 ('P7', 'P9', 'O1'), G8 ('P8', 'P10', 'O2'), G9 ('PZ', 'P3', 'P4 '). This division served two purposes. First, the whole brain area was evenly divided into 9 groups on the scalp (**Figure 1A**). Second, regardless of the EEG equipment, and each subject had at least one EEG channel from each channel group. When there were two or three EEG channels within each group, we averaged the data across these channels. Additionally, we further standardized the temporal information. Due to different sampling rates used in various EEG recordings, we resampled all EEG signals with 250 Hz. In total, we computed 6×9=54 (frequency×group) power ratio features per subject.

For ECG signals, we computed a total of 24 linear and nonlinear features for heart rate variabilty (HRV) ^1,2^:

*Time-domain features*

| mean_nni  sdnn  sdsd  rmssd  median_nni  cvsd  cvnni  mean_hr  max_hr  min_hr  std_hr | Mean of consecutive R-R intervals  Standard deviation of all consecutive normal R-R intervals  Standard deviation of differences between R-R intervals  Square root of the mean of the sum of the squares of differences between adjacent normal R-R intervals  Median of absolute values of successive differences of R-R intervals  RMSSD/mean_nni  Coefficient of variation, sdnn/mean_nni  Mean of heart rate  Maximum of heart rate  Minimum of heart rate  Standard deviation of heart rate |
| --- | --- |

*Frequency-domain features*

| vlf  lf  hf  lf_hf_ratio  lfnu  hfnu  total_power | Very low frequency (0-0.04 Hz) parameter  Low frequency (0.04-0.15 Hz) HRV parameter  High frequency (0.15-0.4 Hz) HRV parameter  LF/HF ratio  LF power of HRV expressed in normal units  HF power of HRV expressed in normal units  Total spectral power |
| --- | --- |

*Nonlinear-domain features*

| csi  cvi  Modified_csi sd1  Sd2  Ratio_sd2_sd1 | Cardiac sympathetic index  Cardiac vagal index  Modified CSI  Standard deviation along the minor axis of Poincare plot  Standard deviation along the major axis of Poincare plot  SD2/SD1 ratio |
| --- | --- |

**Details of CNN training and testing**

For the CNN architecture, we used one stream that received *N*-channel raw EEG time series input with *W*-s duration (downsampled at 200 Hz; resulting in a sample size of 200×*W*). For each stream, we had a set of one-dimensional convolution filters with finite filter length, thereby generating a set of convolution filters (wth size of *m*×*N*). Specifically, the *j*-th (*j*=1,···, *m*) convolution filter $f_{c}^{(j)}$had a parametric form as a product of cosine function and Gaussian-shape kernel ^3^

$$f_{c}^{(j)}\left( \tau\right)=A_{c}^{(j)}\cos\left( \omega^{(j)}\tau+\phi_{c}^{(j)} \right)exp(-\beta_{c}^{(j)}\tau^{2})$$

where the frequency $\omega^{(j)}$ and the precision parameter $\beta_{c}^{(j)}>0$were shared across EEG channels (indexed by *c*), which resembled he real part of a complex Morelet wavelet. In our application, we have tested *m*=2-4 and used a smallest size *m*=2 for better interpretability. These two parameters defined the spectral propeties of the filter, where $\omega^{(j)}$ controled the center of frequency and $\beta_{c}^{(j)}$ controlled the time-frequency resolution tradeoff. The amplitude $A_{c}^{(j)}>0$and phase shift $\phi_{c}^{(j)}$were defined for the *j*-th filter at the *c*-th channel (or channel group). The output of each convolution filter was a filtered signal acrossed all *N* channels

$$h^{(j)}=\sum_{c=1}^{N} f_{c}^{(j)}\left( t \right)\bigotimes x_{c}(t)$$

The filtered output was passed by a rectified linear unit (ReLU), followed by a max pooling operation. Finally, the feature was flattened, concatenated and sent to the the output layer, which computed a softmax function to produce a prediction score beween 0 (non-SUDEP) and 1 (SUDEP).

To construct the training samples, we specified a window size to create non-overlapping independent “snippets” from each subject’s 5-min interictal sleep EEG recording. The cross-entropy loss function was used to assess the CNN training convergence

$$L=\sum_{k=1}^{Batch size} -\left[ y_{k}\log\hat{y}_{k}+(1-y_{k})log(1- \hat{y}_{k}) \right]$$

Where $y_{k}$and $\hat{y}_{k}$denote the target and predicted value for the *k*-th training sample, respectively. We used the batch size of 128.

To train the CNN, we used the Adam algorithm to perform gradient descent optimization ^4^, using a learning rate parameter of 0.1. To avoid overfitting, we also adopted the dropout strategy (probability 0.5) to randomly select the percentage of channels.

During testing, we again used a non-overlapping sliding window and fed multichannel EEG time series into the trained CNN. We computed the prediction scores in the consecutive windows, and then computed an average score by temporal smoothing across multiple sliding windows.

**Selection and impact of window duration**

The choice of temporal window duration reflected the timescale of interest for the multichannel EEG features. During sleep, EEG oscillations are dominated by slow frequency (<40 Hz). We systematically optimized the window duration to achieve the best performance.

**Visualization and interpretation of convolution filters**

To visualize the learned spatiospectral features, we mapped the *m*×*N* convolutional filters onto *m* brain topographies of spatial patterns ^3^. Each filter consisted of a spatial pattern of learned amplitude {$A_{c}^{(j)}\}$and phase shift ${\{\phi}_{c}^{(j)}\}$, with a specific center frequency $\omega^{(j)}$. For the spatial patterns of “amplitude map”, a large value indicates the importance at specific channel or brain region. For the “phase shift map”, a value beween 0 and $\pi$indicates relative phase leading, whereas a value between $\pi$ and $2\pi$ indicates relative phase lagging.

**Discussion**

The parametric CNN model used in the curent paper is motivated from the convolutional neural network (CNN) and deep learning ^3,5^. Recently, CNNs and recurrent neural networks (RNNs) have been adopted to learn multichannel EEG representations ^6,7^, in temporal, spectral and spatial domains. Several deep learning models have been proposed in the literature for epilepic EEG signal classificaiton ^8-11^, but none of them focused on interictal EEG recordings. As most deep learning models have a large number of model parameters, it is subject to overfitting in the presence of a small training sample size. One effective strategy is to impose a sparsity constraint on the parameterized convolutional filter structure onto the deep learning model ^3,12^. Additionally, available methods can be applied to assess the importance or sensitivity of features for the outcome of the deep learning models ^13^. With an increasing number of training sample size, the CNN model can potentiall improve the online risk assessment based on interictal EEG during sleep, while offering interpretable neural signatures extracted from the convolutional filters. Finally, explainable AI and explainable deep learning may provide an interface for neurologogists to uncover the state of “epileptic brain”.

**Supplementary References**

1. Heart rate variability analysis. <https://pypi.org/project/hrv-analysis/>. Open source software: <https://github.com/Aura-healthcare/hrv-analysis>
2. Ponnusamy A, Marques JLB, Reuber M. Comparison of heart rate variability parameters during complex partial seizures and psychogenic nonepileptic seizures. *Epilepsia* 2012; 53(8): 1314-1321.
3. Li Y, Murias, Major S, Dawson G, Dzirasa K, Carin L, Carlson DE. Targeting EEG/LFP synchrony with neural nets. *Adv. Neural Info. Proc. Syst (NeuroIPS’17*), Long Beach, CA.
4. Kingma DP, Ba J. Adam: A method for stochastic optimization. arXiv preprint. 2014; arXiv:14126980.
5. LeCun Y, Bengio Y, Hinton G. Deep learning. *Nature* 2015; 521: 436-444.
6. Bashivan P, Rish I, Yeasin M, Codella N. Learning representations from EEG with deep recurrent-convolutional neural networks. *Proc. Inter. Conf. learned Representations (ICLR’16*). <https://arxiv.org/abs/1511.06448>
7. Stober S, Sternin A, Owen AM, Grahn JA. Deep feature learning for EEG recordings. *Proc. International Conf. learned Representations (ICLR’16*). <https://arxiv.org/abs/1511.04306>
8. Zhu C, Kim Y, Jiang X, Lhatoo S, et al. A lightweight convolutional neural network for assessing an EEG risk marker for sudden unexpected death in epilepsy. *BMC Medical Informatics and Decision* *Making* 2020; 20: 329.
9. Gao Y, Gao B, Chen Q, et al. Deep convolutional neural network-based epileptic electroencephalogram (EEG) signal classification. *Front. Neurosci.* 2020; 11: 375.
10. Acharya UR, Oh SL, Hagiwara Y, et al. Deep convolutional neural network for the automated detection and diagnosis of seizure using EEG signals. *Computers in Biology and Medicine* 2018; 100: 270-278.
11. Lawhern VJ, Solon AJ, Waytowich NR, et al. EEGNet: A compact convolutional network for EEG-based brain-computer interfaces. *J. Neural Eng.* 2018; 15: 056013.
12. Mocanu, D.C., Mocanu, E., Stone, P. *et al.* Scalable training of artificial neural networks with adaptive sparse connectivity inspired by network science. *Nat. Commun.* 2018; 9: 2383.
13. Lapuschkin S, Wäldchen S, Binder A, Montavon G, Samek W, Müller K-R. Unmasking Clever Hans predictors and assessing what machines really learn. *Nat. Commun.* 2019;10(1):1096.

**TABLE S1: Number of SUDEP and control epilepsy patients from eight centers and leave-one-center-out performance**

| **Center** | **# SUDEP**  **at-risk** | **# non-SUDEP controls** | **AUC & Accuracy (LR classifier)** | | |
| --- | --- | --- | --- | --- | --- |
|  |  |  | (iii)  (n=83) | (i)  (n=76) | (i)+(ii)  (n=70) |
| Melbourne (RMH) | 5 (3 F) | 8 (4 F) | 0.72 [0.68,0.76]  0.60 [0.50,0.60] | 0.56 [0.44, 0.56]  0.50 [0.50,0.67] | 0.50 [0.00, 0.50]  0.25 [0.25 0.25] |
| Melbourne (Austin) | 6 (3 F) | 12 (6 F) | 0.64 [0.61,0.72]  0.58 [0.50,0.67] | 0.78 [0.75, 0.81]  0.75 [0.67,0.75] | 0.96 [0.92, 1.00]  0.90 [0.80, 0.90] |
| Melbourne (St. Vincent) | 2 (1 F) | 4 (2 F) | 0.50 [0.50,0.50]  0.25 [0.25,0.50] | 1.00 [1.00, 1.00]  0.50 [0.50, 1.00] | 1.00 [1.00, 1.00]  0.50 [0.50, 0.50] |
| Columbia University | 7 (4 F) | 14 (8 F) | 0.72 [0.67,0.78]  0.67 [0.58, 0.75] | 0.69 [0.64, 0.72]  0.67 [0.58, 0.75] | 0.69 [0.64, 0.72]  0.67 [0.67, 0.75] |
| New York University | 2 (0 F) | 4 (0 F) | 0.75 [0.75,1.00]  0.75 [0.50, 0.75] | 0.75 [0.75, 1.00]  0.75 [0.50, 0.75] | 1.00 [0.75, 1.00]  0.75 [0.50, 0.75] |
| Yale University | 5 (2 F) | 10 (4 F) | 0.36 [0.28,0.44]  0.40 [0.30, 0.40] | 0.80 [0.72, 0.84]  0.70 [0.60, 0.70] | 0.72 [0.68, 0.76]  0.60 [0.50, 0.60] |
| Johns Hopkins University | 1 (0 F) | 2 (0 F) | 0.75 [0.75,1.00]  0.50 [0.50, 1.00] | 1.00 [0.00, 1.00]  0.50 [0.00, 0.50] | 0.00 [0.00, 0.00]  0.00 [0.00, 0.00] |
| University of Cincinnati | 2 (1 F) | 4 (2 F) | 1.00 [1.00,1.00]  0.75 [0.50, 0.75] | 0.75 [0.75, 1.00]  0.75 [0.50, 0.75] | 1.00 [1.00, 1.00]  0.75 [0.75, 0.75] |
| **Total** | 30 (14 F) | 58 (26 F) | **Mean: 0.68, 0.56** | **Mean: 0.79,0.64** | **Mean: 0.73,0.55** |

**TABLE S2: Comparison of three machine learning classifiers in leave-one-center-out AUC using features (i)+(ii)**

| **Center** | **# Total tested subjects**  **(SUDEP+control)** | **AUC**  **(SVM classifier)** | **AUC**  **(RF classifier)** | **AUC**  **(LR classifier)** |
| --- | --- | --- | --- | --- |
| Melbourne (RMH) | 2+5 | 0.50 [0.25, 0.50] | 0.50 [0.25, 0.50] | 0.50 [0.00, 0.50] |
| Melbourne (Austin) | 5+12 | 0.88 [0.80, 0.96] | 0.76 [0.66, 0.88] | 0.96 [0.92, 1.00] |
| Melbourne (St. Vincent) | 1+1 | 1.00 [1.00, 1.00] | 0.00 [0.00, 0.00] | 1.00 [1.00, 1.00] |
| Columbia University | 6+13 | 0.69 [0.61, 0.72] | 0.64 [0.60, 0.71] | 0.69 [0.64, 0.75] |
| New York University | 2+4 | 1.00 [0.75, 1.00] | 1.00 [0.75, 1.00] | 1.00 [0.75, 1.00] |
| Yale University | 5+8 | 0.71 [0.64, 0.76] | 0.56 [0.46, 0.60] | 0.72 [0.68, 0.76] |
| Johns Hopkins University | 1+1 | 0.00 [0.00, 0.00] | 0.00 [0.00, 1.00] | 0.00 [0.00, 0.00] |
| University of Cincinnati | 2+2 | 1.00 [1.00, 1.00] | 0.75 [0.75, 0.75] | 1.00 [1.00, 1.00] |
| **Total** | 24+46 = 70 | **Mean: 0.723** | **Mean: 0.526** | **Mean: 0.734** |

**TABLE S3: Statistics of misclassified subjects with depression or developmental delay/static** **encephalopathy**

|  | **Depression** | **Developmental Delay/Static Encephalopathy** |
| --- | --- | --- |
| False Positive | 16.7% | 33% |
| True Positive | 6.7% | 40% |
| True Negative | 33% | 18% |

**Figure S1. Comparison of ECG heart rate variability (HRV) statistics between SUDEP patients and age-matched living epilepsy controls**. (**A**) lfnu. (**B**) hfnu. (**C**) lf. (**D**) lf/hf ratio. (**E**) min_hr. (**F**) std_hr.

**Figure S2. Schematic flowchart of EEG/ECG data analytics in SUDEP risk assessment.**

**Figure S3. Schematic diagram of convolutional neural network (CNN) for sleep EEG spatiospectral feature extraction, as applied to sliding-window based SUDEP risk assessment.** During sleep, multichannel EEG signals were fed to the CNN, which consists of convolution filters, max pooling and flatten operations. These spatiospectral EEG features (mapped onto a brain topography of spatial patterns in heat map) were further sent to a fully connected layer to compute a predictive score between 0 and 1 in the softmax output layer. At the final decision stage, temporal smoothing was applied to the sliding predictive scores to produce a SUDEP risk assessment.

**
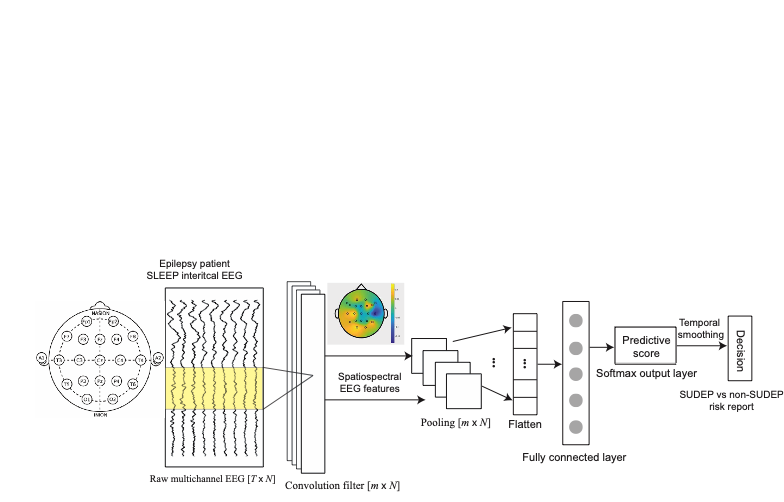
**

**Figure S4. Cross-validated AUC statistics with the number of selected EEG+ECG features**. The error bar denotes the SD based on 1000 Monte Carlo runs in the *L*_1_ regularized LR classifier.

**Figure S5. Mean regression coefficients associated with EEG and ECG features used in *L*_1_ regularized LR classifier.**

**Figure S6.** **Impact of sliding window duration on online classification performance**. In each condition, the box plot statistics were computed based on Monte Carlo runs (n=1000 in LR, and n=100 in CNN).


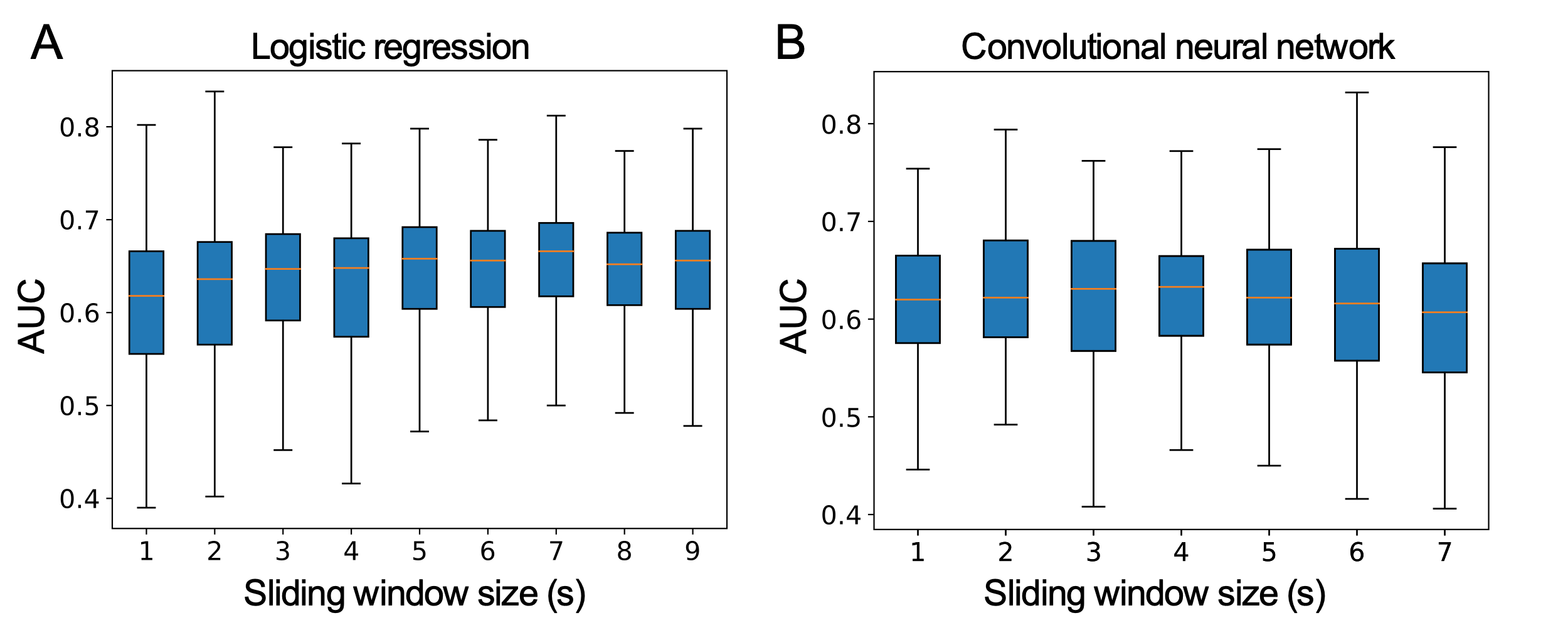


**Figure S7. Classification performance was stable in the presence of sleep EEG signal non-stationarity**. ROC curves were computed based on training the first half of sleep EEG data and testing the second half of sleep EEG data. Diagonal line indicates the chance level.
